# Thyroid Hormone and Seasonal Rhythmicity

**DOI:** 10.3389/fendo.2014.00019

**Published:** 2014-02-26

**Authors:** Hugues Dardente, David G. Hazlerigg, Francis J. P. Ebling

**Affiliations:** ^1^Physiologie de la Reproduction et des Comportements, INRA, UMR085, Nouzilly, France; ^2^CNRS, UMR7247, Nouzilly, France; ^3^Université François Rabelais de Tours, Tours, France; ^4^Institut français du cheval et de l’équitation, Nouzilly, France; ^5^Department of Arctic and Marine Biology, University of Tromsø, Tromsø, Norway; ^6^School of Life Sciences, University of Nottingham, Nottingham, UK

**Keywords:** seasonality, reproduction, pars tuberalis, melatonin rhythm, kisspeptins, RF-amide, GnRH neurons

## Abstract

Living organisms show seasonality in a wide array of functions such as reproduction, fattening, hibernation, and migration. At temperate latitudes, changes in photoperiod maintain the alignment of annual rhythms with predictable changes in the environment. The appropriate physiological response to changing photoperiod in mammals requires retinal detection of light and pineal secretion of melatonin, but extraretinal detection of light occurs in birds. A common mechanism across all vertebrates is that these photoperiod-regulated systems alter hypothalamic thyroid hormone (TH) conversion. Here, we review the evidence that a circadian clock within the pars tuberalis of the adenohypophysis links photoperiod decoding to local changes of TH signaling within the medio-basal hypothalamus (MBH) through a conserved thyrotropin/deiodinase axis. We also focus on recent findings which indicate that, beyond the photoperiodic control of its conversion, TH might also be involved in longer-term timing processes of seasonal programs. Finally, we examine the potential implication of kisspeptin and RFRP3, two RF-amide peptides expressed within the MBH, in seasonal rhythmicity.

## Introduction

Seasonality is a critical property of most organisms. At temperate latitudes, photoperiod is the main synchronizer of seasonal functions. Photoperiodism defines the use of the annual cycle of day and night length to coordinate functions such as reproduction, fattening, hibernation, and migration with predictable changes in the environment, for example in food availability or climatic conditions. Seasonal changes in physiology and behavior typically are innately timed long-term processes, requiring weeks or months to wax and wane. Therefore, additional to photoperiodic readout mechanisms, living creatures have evolved endogenous long-term timing devices, which allow them to anticipate forthcoming seasonal changes. In the most extreme cases, cycles of about 365 days recur for years in animals kept under constant photoperiods; such so-called circannual rhythms exist in a variety of birds and longer-lived mammals.

Species with relatively short life spans such as voles and hamsters usually do not display circannual rhythms, but their seasonal cycles also comprise an endogenously generated part, which corresponds to the overwintering period and allows timely emergence from the burrow and reproductive recrudescence in early spring. Endogenous long-term timing is commonplace in vertebrates but its mechanistic basis remains mysterious [for reviews, see Ref. (1–6)]. Here we review findings, essentially in birds and mammals, which clarify the mechanisms of photoperiodic readout and provide a rationale for the seasonal control of thyroid hormone (TH) metabolism within the hypothalamus.

## Photoperiodism: Melatonin and the Pars Tuberalis

The crucial role of melatonin in mammalian photoperiodism has been established in many species including hamsters, ferrets, and sheep (7–9). Within the pineal, melatonin is produced and released during the night and therefore constitutes an internal neurochemical representation of photoperiod. Timed melatonin-infusion experiments established that duration is the key parameter of the melatonin pattern that triggers the photoperiodic response [for review, see Ref. (10)]. In order to map central binding sites, autoradiography with 2-iodo-melatonin was used in a wide range of mammals (11). Surprisingly, across all species the highest density of melatonin-binding sites was found in the *pars tuberalis* (PT), a region of the pituitary stalk apposed to the median eminence. The suprachiasmatic nuclei (SCN) also showed moderate labeling in most species while many brain nuclei showed weak to moderate labeling, with very little species overlap [for reviews, see Ref. (12, 13)]. The presence of melatonin receptors within the SCN was consistent with the effects of melatonin on daily timing in mammals (14). Conversely, since the PT was the only neuroendocrine structure labeled in the highly photoperiodic ferret, a role in seasonality was anticipated (15). However, melatonin-binding sites were also disclosed within the PT of species, which are not overtly photoperiodic such as mouse, rat, and human.

Melatonin-binding studies also led to the recognition that the binding site(s) for melatonin was a classical GPCR, with picomolar affinity for its ligand. In mammals, two high-affinity melatonin receptors (MT1 and MT2) were cloned (16, 17). Subsequent studies showed that MT1 is the predominant subtype, both necessary and sufficient to mediate the photoperiodic effect of melatonin (18–22). The number of central sites expressing melatonin receptors as revealed by *in situ* hybridization was comparatively more restricted – mostly the PT and the SCN – than that observed with melatonin-binding studies. This may reflect the difference in sensitivity of the techniques and/or the existence of a low-affinity melatonin-binding site. The latter would be physiologically irrelevant, and probably corresponds to quinone reductase 2 rather than a true melatonin receptor (23).

## Melatonin-Dependent TSH Release in the Pars Tuberalis

The PT is the most rostral part of the adenohypophysis. Many reviews detailing the ontogeny, morphology, and immunohistochemical characteristics of the PT are available (24–28). The PT was once considered an “undifferentiated embryological remnant of the hypophysis” whose “only function is to provide mechanical support role for the hypothalamo-hypophyseal portal vessels” [see Ref. (29)]. However, its location and anatomical features pleaded in favor of a specific role: the PT extends along the ventral aspect of the median eminence, surrounds the pituitary stalk in its most caudal part, and is in contact with nerve endings of the median eminence and capillaries of the pituitary primary plexus.

The PT is phylogenetically conserved in tetrapods, but is generally absent in fish (30), and consists of endocrine cells, which exhibit early secretory activity compared to the pars distalis (PD). Three different cell types occur in the PT: (i) follicular cells; (ii) gonadotropes, which constitute ~10% of the endocrine PT cells, have dense-core granules and occur mostly in the caudal PT (known as the *zona tuberalis*); (iii) PT-specific cells, which are virtually agranular thyrotropes and constitute ~90% of endocrine PT cells. The PT gonadotropes appear identical to those in the PD, while shape and ultrastructure of PT-specific thyrotropes differ strikingly from those in the PD (24, 25). These thyrotropes were therefore suspected to be a peculiar pituitary endocrine cell type, possibly producing a novel glycoprotein [“tuberalin,” Ref. (31)]. These cells exhibit early secretory activity compared to PD endocrine cells (32). This depends upon the induction of *Tshβ* transcription by a transcription factor consequently called TEF [Thyrotroph Embryonic Factor; Ref. (33)].

Based on ultrastructure and immunohistochemistry, these PT-specific thyrotropes were predicted to be melatonin-responsive, a prediction which has since been validated (34, 35). TSH immunoreactivity within these cells displays dramatic melatonin-dependent photoperiodic changes, with high and low levels under long (LP) and short photoperiod (SP), respectively (36, 37). Finally, the TSH produced by these PT-specific thyrotropes may be identical to that produced by the PD, but the transcriptional control of the *Tshβ* gene in the two populations differs since PT thyrotropes do not express receptors for either TRH or TH (38). Hence, *Tshβ* expression by PT-specific thyrotropes is disconnected from the classical hypothalamic–pituitary–thyroid axis; instead it depends upon melatonin.

However, considering the Harris dogma of a descending flow of information from the hypothalamus to the pituitary, a role for PT-derived TSH was not forthcoming. Rather, it was assumed that, should the PT play a role in seasonality, it would most probably be to release tuberalin(s) in the pituitary portal plexus, which would then target the PD. This might be the case for the seasonal control of the lactotropic axis, even though the mechanism is unclear (39). This aspect will not be considered further here as it has been discussed elsewhere (28, 40–42).

## Thyroid Hormone Signaling in Seasonal Cycles

### An overview

**Figure 1:**
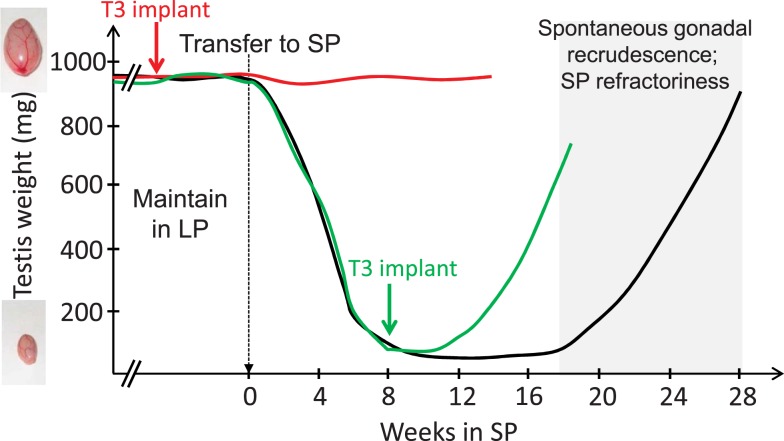
**T3 implants prevent SP-induced inactivation of the gonadal axis (red line) and reactivate the gonadal axis in SP-adapted Siberian hamsters [green line; after data from Barrett et al. (54) and Murphy et al. (55)]**. Siberian hamsters (black line) kept in LP remain indefinitely sexually active (broken lines) unless they are transferred to SP; gonads then progressively regress (testes depicted here, but data are similar for female reproductive organs). However, prolonged SP exposure leads to a spontaneous recrudescence of the gonads, which reflects SP-refractoriness.

The pioneering work of Benoit on ducks in the 1930s revealed that the thyroid gland is mandatory for seasonal transitions in reproductive states, a finding which applies to a wide range of vertebrates [reviewed by Nicholls et al. (43); Hazlerigg and Loudon (44); Yoshimura (45)]. Thyroidectomy prevents the cessation of breeding in starlings (46), quail (47), and sheep [Ref. (43, 48, 49); for review, see Ref. (50)]. In rams, thyroidectomy during the non-breeding season almost immediately reactivates the gonadotropic axis (51). Therefore, TH appeared to transmit the message of long-day lengths. Microimplants releasing small amount of TH were then surgically placed within the brain of the ewe (52, 53), which revealed that TH acts centrally, and most likely within the medio-basal hypothalamus (MBH), to impact seasonal reproduction. Studies in Siberian hamsters using a similar microimplants approach further showed that other seasonal axes are also controlled by central actions of T3: providing T3 directly within the MBH overrides the SP-induced inactivation of the gonadotropic axis (54) and triggers premature gonadal recrudescence in SP-exposed animals. T3 implants also override SP-induced seasonal inappetence, weight loss, and expression of torpor [Ref. (55); see Figure 1]. Similar outcomes are found when T3 is provided by daily subcutaneous injections to SP-exposed hamsters (56). In contrast to these effects on reproduction and energy metabolism, T3 implants do not impact the lactotropic axis, consistent with a distinct mechanism of control (57, 58) while not incompatible with a common melatonin target tissue as discussed later.

These observations added to the well-documented role of TH in key transitions between life cycles, such as metamorphosis in amphibians and developmental growth and differentiation of the mammalian brain (59, 60). In adults, TH also has key roles in the control of metabolism and thermoregulation, two processes intertwined with the seasonal reproductive cycle. The seasonal program encompasses profound and coordinated changes in behavioral, reproductive, and metabolic states (61). The finding that TH regulates the basal metabolic rate is not new, but the recognition that it reflects a central action within the MBH is very recent (62, 63). Indeed, T3 injection within the MBH suffices to promote food intake and weight gain in rats (64). Interestingly, this effect is mimicked by LP, which triggers weight gain in many species, including sheep (65). Such a process bears critical adaptive value, best exemplified in species that hibernate (e.g., groundhog) or undergo daily torpor (e.g., Siberian hamster), which have evolved a strategy to build up abdominal fat depots during spring/summer to survive the harsh winter season (1, 61). Photoperiodic cues and the metabolic status interact in many seasonal breeders, including sheep (66, 67), goats (68, 69), and horses (70). In all these species, feeding modulates the duration of the breeding season and/or depth of the anestrus. Therefore, TH integrates and coordinates physiological changes, which are integral to the seasonal program.

### Local control of TH metabolism within the MBH

Although cold exposure activates thyroid activity, under constant ambient temperature conditions, TH concentrations do not display marked or consistent seasonal fluctuations in the plasma or cerebro-spinal fluid. Rather, fine temporal and local control of TH action is achieved through opposite actions of specific enzymes known as deiodinases (71–73). Deiodinase 2 (DIO2) converts the relatively inactive T4 into the active T3 while deiodinase 3 (DIO3) inactivates T4 by converting it into rT3, and also degrades T3 into T2. Very precise control of T3 concentrations is further achieved through reciprocal control of the expression and activity of these two enzymes by their ligand: a hypothyroid state up-regulates DIO2 and down-regulates DIO3, and vice-versa (72, 74, 75).

The central expression of *Dio2* is restricted to a few structures. The pineal gland is one of them (76), but the strongest expression occurs in astrocytes and tanycytes lining the third ventricle and median eminence (77, 78). These tanycytes also express two major TH transporters, MCT8 and OATP1c1 (79–81), and MCT8 is expressed at higher levels under SP than LP in the Siberian hamster (82, 83). Tanycytes are a heterogeneous and complex population of ependymal cells, which constitute a gateway between the CSF and the MBH and median eminence (84). In a pioneering study, Yoshimura and colleagues (85) showed that both *Dio2* and *Dio3* are expressed within tanycytes of the quail MBH. Crucially, the expression of these two enzymes displays opposite regulation by photoperiod: *Dio2* is highly expressed under LP while *Dio3* is highly expressed under SP. This predicted a local increase of T3 content within the MBH under LP, which was validated by radioimmunoassay (85). The opposite regulation of *Dio2* and *Dio3* by photoperiod has since been described in sparrows and Siberian and Syrian hamsters (86–89). Importantly, the expression of *Dio2* is down-regulated by melatonin, independently of sex steroids (88, 90). Melatonin is also required to trigger *Dio3* expression under SP in Siberian hamster (54). Collectively, these data provided an enzymatic means through which local T3 levels in the MBH could increase under LP.

### Closing the loop: TSH output from the PT governs T3 regulation within the MBH

The PT seemed well located to mediate photoperiodic switches in *Dio2*–*Dio3* usage. To decipher the mechanism of the photoperiodic response, Yoshimura and colleagues (91) set out an ambitious experimental set-up: hypothalamic blocks containing the MBH and PT/median eminence from quails submitted to a long-day transfer, known to activate the gonadotropic axis within 24 h, were used for hybridization on a chicken gene chip. This revealed that the expression of two genes, *Tshβ* and *Eya3*, is rapidly triggered by the transfer from SP to LP. A second wave of transcriptional changes was also observed for a handful of genes including *Dio2* and *Dio3*, which displayed acute and simultaneous induction and repression, respectively. Crucially, expression of the cognate TSH receptor (TSHR) was found in tanycytes, which express the deiodinases, providing the link between TSH output from the PT and T3 regulation within the MBH. The pathway was uncovered using an acute intracerebroventricular injection of TSH to SP-exposed quails, which induced *Dio2* expression and led to gonadal recrudescence.

In a contemporaneous study in sheep, Hanon et al. (92) suggested this mechanism to be ancestral, since their data were similar in many respects: higher *Tshβ* expression within the PT under LP than SP (see Figure 3A), expression of the TSHR within tanycytes and PT/median eminence, higher *Dio2* expression under LP than SP (see Figure 3A), and TSH-dependent induction of *Dio2* both *in vitro* and *in vivo*. The latter finding was not unexpected, since TSHR signals through a Gs protein, and *Dio2* is a cAMP-responsive gene (93). In contrast, the MT1 receptor couples to a Gi protein and the interplay between TSHR and MT1 signaling within the PT may be part of the photoperiod decoding mechanism, at least in sheep (92, 94, 95). Under LP, the PT therefore functions as an “indirect T3-generator,” disconnected from both TRH and T3 feedback (see above).

**Figure 2:**
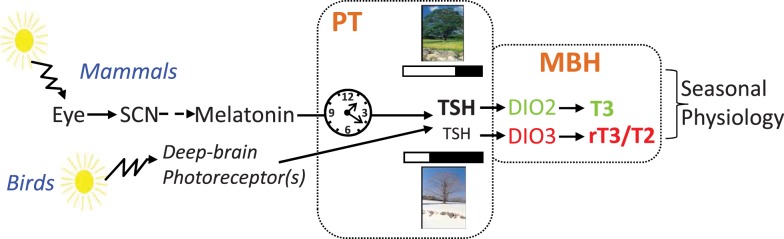
**Pathways for photoperiodic entrainment in mammals and birds (see text)**.

Since these studies in quail and sheep, a similar TSH/deiodinases/T3 retrograde pathway (from the pituitary back to the hypothalamus, Figure 2) has been described not only in other photoperiodic species such as the European hamster (96), the Syrian hamster (97), the Siberian hamster (89), the common vole (98), but also in photoresponsive juvenile Fisher 344 rats (99) and in a melatonin-producing but non-photoperiodic CBA/N mouse strain (22, 100, 101). Photoperiodic variations in *Dio2* expression were however not observed in the non-photoperiodic Wistar rat (88). The use of murine knock-out strains confirmed that the MT1 melatonin receptor (22) and TSHR (100) are mandatory for the LP induction of *Dio2* expression within tanycytes. Whether this pathway is present in all vertebrates remains to be determined (102).

As mentioned before, fish species investigated thus far do not have a distinct PT, but in masu salmon a TSH/DIO2 axis implicating the *saccus vasculosus*, located below the hypothalamus and caudally to the pituitary gland, has been proposed (103). However, the *saccus vasculosus* is absent in several species of fish such as the pike (104), which is nonetheless photoperiodic (105). The other few studies on this matter in fish have yielded varied outcomes (106, 107). Regarding birds, studies in tits (108) and starlings (109) did not lend clear support to the model, but aspects of the experimental set-up prevent any conclusion to be drawn. For example, the studies of starlings were carried out in outdoor aviaries, so effects of fluctuating temperature on the peripheral thyroid axis may have obscured the photoperiodic regulation of DIO2 and DIO3 centrally. Finally, we are not aware of any study on this matter in either reptiles or amphibians.

## Encoding and Decoding the Photoperiodic Message

### Upstream of the PT

Birds and mammals possess a similar mechanism to respond to photoperiod, but they perceive the photoperiodic message in different ways. In mammals, light is exclusively perceived by the retina, with a key role for ganglion cells expressing the photopigment melanopsin [for review, see Ref. (110)]. This information is relayed to the circadian clock of the SCN, which governs melatonin production by the pineal gland through a multi-synaptic sympathetic pathway. Melatonin is the mandatory messenger of photoperiod in mammals. In striking contrast, removing the eyes and suppressing melatonin by pinealectomy does not disrupt photoperiodism in birds [for reviews, see Ref. (44, 45, 111–113)]. In birds, light goes through the skull and acts directly upon hypothalamic deep-brain photoreceptors to control seasonal reproduction (Figure 2). Several photopigments expressed by different cell types, all located within the MBH and projecting to the PT/median eminence, are plausible candidates: VA-opsin (114), neuropsin [Opn5, Ref. (115, 116)], and melanopsin [Opn4, Ref. (117)]. The neurotransmitter(s) and/or neuropeptide(s) used by these cells, and how they impinge on PT thyrotropes, remain to be elucidated.

### Within the PT: From the circadian clock to the seasonal output

Photoperiodic species such as quail (118) and Siberian and Syrian hamsters (119, 120) measure photoperiod length with remarkable accuracy. In these three species, reproduction switches off when the photoperiod is shorter than 12.5 h. The narrow photoperiod range over which physiological changes occur is one of the lines of evidence implicating some sort of daily timing device. The concept that circadian clock(s), clocks with a period of about 24 h, control seasonal timing is indeed not novel (120, 121).

The genetic and molecular bases and organization of circadian clocks have been recently identified (122–124). These clocks are not only located within the SCN, but are present in virtually every tissue and cell where they impact “local” physiology. The PT is no exception as it expresses a full set of clock genes and displays persistent circadian rhythmicity *in vitro* [Ref. (125–127); for review, see Ref. (28)]. The SCN and peripheral clocks share fundamental characteristics: they are cell-autonomous and self-sustained. However, individual cellular clocks within peripheral tissues rapidly become desynchronized and exhibit phase drifting in the absence of regular resetting by cues emanating, directly or indirectly, from the SCN. These cues include inputs from the autonomic nervous system, temperature cycles, and humoral factors such as glucocorticoids and melatonin.

**Figure 3:**
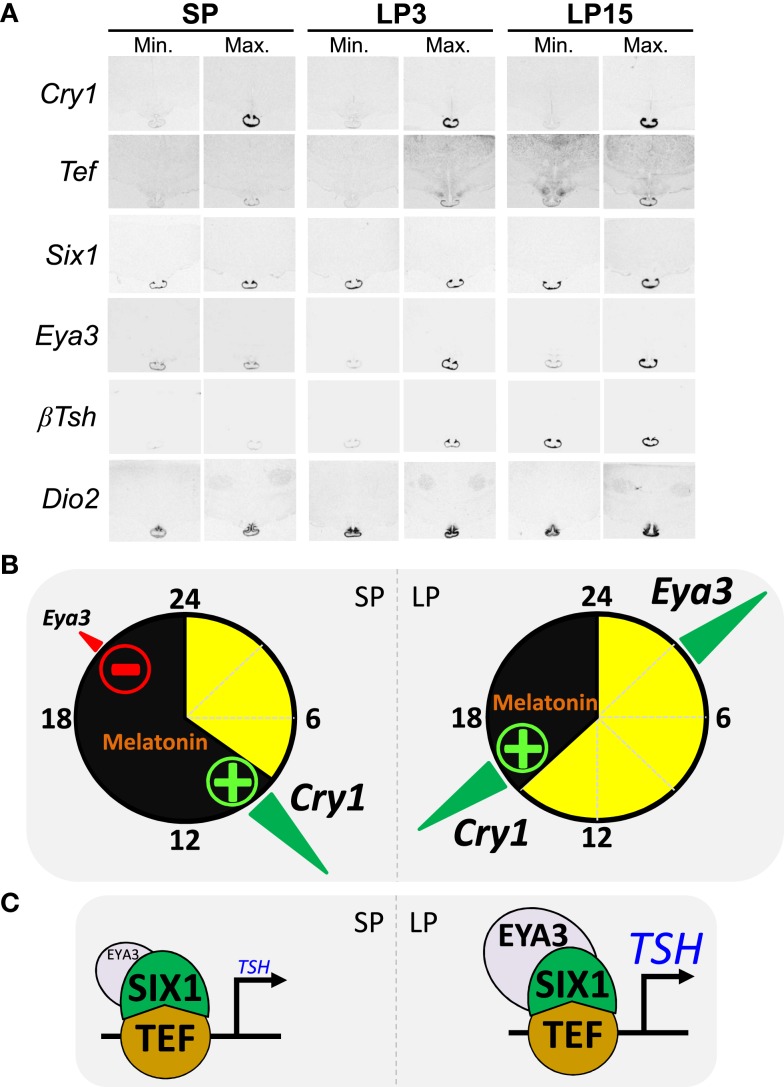
**The circadian clock of the *pars tuberalis* links melatonin to the photoperiodic response [after data from Dardente et al. (138) and unpublished data]**. **(A)** Images representative of minimal and maximal mRNA levels *in situ* hybridization autoradiograms for *Cry1, Tef*, *Six1, Eya3, Tshβ*, and *Dio2* in sheep kept under SP 8:16 and sheep transferred to LP 16:8 for 3 days (LP3) or 15 days (LP15). **(B)** The internal coincidence model for photoperiodic time-measurement within the PT; SP situation on the left side, LP on the right side, yellow and black indicate day and night. The transcription of *Eya3* is both clock-controlled and inhibited by melatonin, hence the phase-relationship relative to *Cry1* expression (a melatonin-induced circadian gene) is similar irrespective of the photoperiod but *Eya3* transcription increases only under LP as melatonin inhibition is relieved. **(C)** Schematics of the transcriptional control of the *Tshβ* gene by TEF/SIX1/EYA3. Note that EYA3 levels are higher under LP than SP.

The PT can be defined as a melatonin-dependent circadian oscillator (28, 50). Resetting of the PT clock by melatonin requires acute induction of *Cry1* expression [Ref. (128, 129), see Figure 3A]; CRY1 being a key repressor of the circadian clock (130–132). The acute induction of *Cry1* expression involves EGR1-like factors (133) and the transcription factor NPAS4 (134, 135). In sheep, *Cry1* expression remains tightly linked to the onset of melatonin secretion and by implication night onset, irrespective of the duration of the day length [Ref. (136), see Figure 3B]. Interestingly, light given during the night induces *Cry1* expression within the quail PT (137), which suggests a phylogenetically conserved role for *Cry1* in the photoperiodic resetting of the PT clock.

How do we connect melatonin resetting of the PT clock with differential photoperiodic output of TSH and seasonal reproduction? The expression of the transcriptional co-activator EYA3 within the ovine PT displays large photoperiodic changes in both phase and amplitude [Ref. (39, 138); see Figure 3A]. Interestingly, *Eya3* was the other gene (besides *Tshβ*) immediately induced in the quail PT during the first long-day release experiment (91). We therefore investigated the transcriptional control of *Eya3* and searched for a link between inductions of both genes. The expression of *Eya3* is clock-controlled, through conserved DNA binding motifs within its promoter, and therefore phase-locked to that of the circadian clock [Ref. (138), see Figure 3B]. Because of this, expression peaks during the night under SP but during the day under LP. The amplitude of the peak is higher under LP than SP because melatonin suppresses *Eya3* expression, a suppression which can only occur in SP-exposed animals [Ref. (138); see Figure 3B]. Finally, *in vitro* data showed that induction of *Tshβ* expression is triggered by the circadian-controlled transcription factor TEF (33), which then recruits the co-activators SIX1 and EYA3. This leads to a marked increase in transcription under LP due to higher levels of EYA3 [Ref. (102, 138), Figures 3B,C]. A critical role for SIX1/EYA3, but not TEF, in the photoperiodic control of *Tshβ* transcription in the mouse PT has been proposed (139, 140).

### Is T3 output sufficient to elicit the full spectrum of seasonal changes?

The data reviewed so far are consistent with a crucial role for the TSH output of the PT in driving seasonal changes in T3 availability within the MBH. However, swings in TSH/T3 may not be sufficient to elicit all seasonal changes. As mentioned before, since control of the lactotropic axis does not depend on T3 [for review, see Ref. (50)], complementary mechanisms are indeed expected. Neuromedin U (89, 141), histamine, and VGF secretion (82, 142, 143) may mediate seasonal effects on body weight and metabolism since their synthesis and cognate receptors display expression patterns and seasonal changes reminiscent of those seen for TSH/TSHR. However, since TSH infusion in SP-adapted Siberian hamster restores hypothalamic expression of somatostatin and body weight to LP levels (144), Neuromedin U, histamine, or VGF may be dispensable.

Retinoic acid signaling is also likely to be involved as retinoic acid receptors, transporters, and associated binding proteins display prominent photoperiodic regulation in the ependymal cell layer and posterior arcuate nucleus of Siberian hamsters and juvenile Fischer F344 rats (142, 145, 146). Interestingly, the retinoic X receptor (RXR) can heterodimerize with either the TH receptors (THRα/THRβ) or the retinoic acid related receptor (RAR). The target genes and downstream pathways governed by THR and RAR diverge, and therefore the photoperiodic regulation of RXR/RAR may fine-tune the seasonal adaptation of the metabolic status. From a more general standpoint, the notion that tanycytes coordinate a host of seasonal neuroendocrine cycles including reproduction, metabolism, and hibernation is emerging rapidly [Ref. (147, 148); for reviews, see Ref. (61, 149, 150)].

## Photoperiodic Timing and the Circannual Clock: T3 as a Unifying Component?

**Figure 4:**
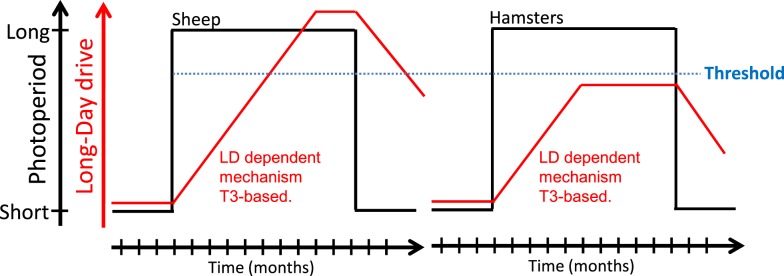
**A model for long-day refractoriness [adapted from Figure 2 in Ref. (152)]**. In sheep (left panel) and hamsters (right panel), exposure to long days (black line) leads to the development of a mechanism of unknown nature, most likely T3-dependent (red line). In sheep, the long-day drive eventually exceeds a “threshold” (blue dotted line); the animal then becomes refractory to long days and spontaneously reverts to an SP phenotype. In hamsters, the long-day drive never exceeds the threshold and the animal displays the LP phenotype indefinitely; exposure to SP is mandatory to get the SP physiological state.

As mentioned earlier, whether species are classified as photoperiodic (e.g., Siberian and Syrian hamsters) or circannual (e.g., sheep), part of the seasonal cycle is generated endogenously. Hamsters and sheep maintained under constant SP do spontaneously revert to the opposite physiological state after several months. This phenomenon, referred to as “SP refractoriness,” is typical of an interval timer/hourglass (5, 151). In contrast, sheep but not Siberian or Syrian hamsters, also display refractoriness to LP. Whether this species difference reflects fundamentally divergent underlying mechanisms is questionable. Indeed, Follett and Nicholls (47) proposed years ago that “it may well be that essentially identical physiological mechanisms underlie the photoperiodic responses of a wide range of vertebrates and that very minor modifications of these can cause surprisingly large (though superficial) changes in the overt responses of the animal in terms of reproduction.” These authors devised a model, based on differences in threshold sensitivity, which rationalizes the LP refractoriness process (see Figure 4). There are indeed similarities between the photoperiodic control of the seasonal program in photoperiodic and circannual species (43, 50, 152). Siberian or Syrian hamsters and sheep might therefore exemplify “variations on a theme” rather than fundamentally different models.

Because TH is involved in many long-term life cycles events, it seems plausible that photoperiod-induced changes in T3 levels may also trigger more profound long-term changes, culminating weeks to months later. In particular, TH-induced plasticity and cell-cycle related events have long time constants, which appear compatible with seasonal cycles (6, 153). Recent data in sheep demonstrate a photoperiodic gating of cell division within the PT and ependymal cells of the 3V and are consistent with this scheme (154–156). Nevertheless, whether photoperiodic gating of cell division depends on TH and/or is involved in seasonal transitions remains to be established.

**Figure 5:**
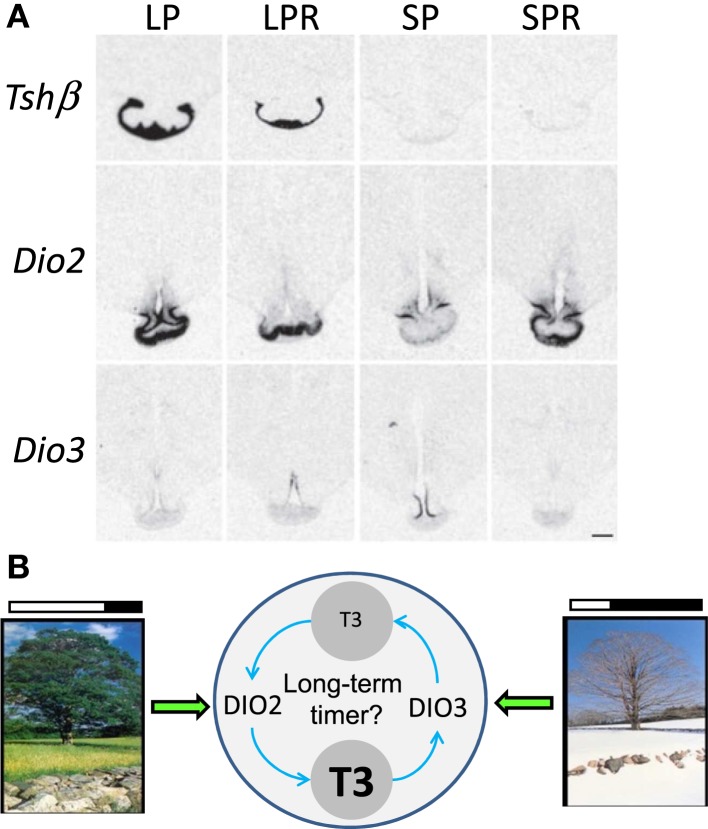
**Beyond the long-day response: TH metabolism within the MBH in long-term timing**. **(A)** Representative images of *in situ* hybridization autoradiograms for *Tshβ, Dio2*, and *Dio3* in sheep under four different endocrine states: LP animals in a spring/summer-like state of reproductive arrest, LP refractory (LPR) animals showing spontaneous reproductive reactivation (late summer/autumn state), SP animals showing autumn/winter-like reproductive activation, and SP refractory (SPR) animals showing spontaneous reproductive arrest [adapted from Saenz de Miera et al. (157)]. **(B)** Schematics depicting (i) the direct effect of LP and SP on DIO2/DIO3 levels, respectively, intertwined with (ii) the possibility that their activity and the resulting TH metabolism constitutes the core of a long-term timing mechanism involved in refractoriness.

To address a potential role for TH turn-over beyond the photoperiodic response, we investigated the expression of *Tshβ* and *Dio2*/*Dio3* within the MBH of sheep under distinct physiological states: LP, LP refractory (LPR) obtained after prolonged LP exposure, SP and SP refractory (SPR) obtained after prolonged SP exposure (157). The expressions of *Tshβ* and *Dio2* were diminished in LPR compared to LP animals but remained low in SP and SPR animals. The expression of *Dio3* was high in SP but very low in all other photoperiodic conditions, most notably under SPR; so the expression of *Dio3* under SP is transient (see Figure 5A).

Therefore, a diminished TSH output may cause the LPR state, while development of the SPR state would be disconnected from it. This would be consistent with the hourglass properties of the SPR mentioned before. However, changes in *Dio2*/*Dio3* may reflect an indirect effect of photoperiod: within the MBH, the local hyperthyroid state triggered by persistent LP exposure would eventually cross a certain threshold, thereby triggering *Dio3* induction and *Dio2* down-regulation (72, 74, 75). Following this, T3 levels would be cleared by DIO3, ultimately leading to the demise of *Dio3* expression; LP exposure would then somehow be required to induce *Dio2* once more and prime a new cycle. This LP requirement to prime the seasonal sequence may explain why circannual cycles in sheep are most obvious under constant LP (50).

Interestingly, Syrian hamsters in SPR state do not exhibit spontaneous reactivation of *Dio2* expression (88) while Siberian hamsters do (83). Furthermore, Siberian hamsters express *Dio3* upon transfer from LP to SP but its expression is not sustained through time (54, 83, 87), similar to what occurs in sheep (157). Therefore, transient *Dio3* expression under SP appears as a common feature and may explain why *Dio3* expression has not been observed in Syrian hamster (54). Even though the relative variations of *Dio2* and *Dio3* differ between species (e.g., Siberian vs. Syrian hamster) the central tenet remains the same: T3 levels are higher within the MBH under LP compared to SP (158). Furthermore, TH metabolism within the MBH may not only intervene in the photoperiodic response but may also be integral to longer-term timing processes such as circannual rhythms (see Figure 5B).

## Conclusion

At this stage several outstanding questions remain: first, since the same TSH/deiodinase/T3 pathway is triggered by LP not only in long-day breeders (e.g., hamsters and quail) but also in short-day breeders (e.g., sheep) and non-photoperiodic species (e.g., mouse), how do we get opposite responses, or no response at all, of the hypothalamic–pituitary–gonadal axis? This is particularly intriguing because the increased intra-hypothalamic availability of TH is uniformly linked to an anabolic state across seasonal species. Second, through which mechanisms do local changes of T3 within the MBH ultimately impinge on gonadotropin-releasing hormone neurons? Pertinent to this second question, the MBH hosts two cell populations expressing RF-amide peptides which have attracted particular attention: neurons of the arcuate nucleus, which express *Kiss1* and neurons of the VMH/DMH, which express the *Rfrp* precursor. The concept that these RF-amide peptides are involved in seasonal breeding has been the topic of several excellent reviews (50, 158–161) and we will therefore only briefly review the most recent and salient findings.

Kisspeptin is a very potent GnRH secretagogue and governs most aspects of reproduction in mammals including sexual differentiation, steroid-dependent gonadotropin release, puberty onset, and the control of fertility by metabolic cues (162, 163). Interestingly, the annual onset of fertility in photoperiodic species had been compared to a reoccurrence of puberty, and common underlying processes were anticipated (2, 164, 165). Kisspeptin therefore appeared a prime candidate for the integration of photoperiodic and metabolic cues across the seasonal program, a prediction which has now received strong support (159–161).

In contrast to kisspeptin, the exact role(s) of peptides derived from the *Rfrp* precursor, RFRP1 and RFRP3, remain(s) unclear (160). RFRP3 may modulate feeding and various stress responses (166, 167). In the context of breeding, RFRP3 inhibits GnRH in sheep [Ref. (168), but see Ref. (169)] but inhibits or activates GnRH in Syrian and Siberian hamsters, depending on the photoperiod (170, 171). The *Rfrp* gene is orthologous to avian *GnIH*, which gives rise to gonadotropin inhibitory hormone (GnIH), a peptide with well-characterized inhibitory effects upon the gonadotropic axis in birds (172). Interestingly, there is no avian ortholog of the *Kiss1* (or *Kiss2*) gene (173), which implies that the concept of a balance between KISS1 and RFRP3 in governing GnRH secretion in mammals (174) does not apply to birds.

Both *Kiss1* and *Rfrp* expression display marked photoperiodic, melatonin-dependent, changes in mammals (159, 160, 175). Even though melatonin receptors have been localized to several hypothalamic nuclei it seems likely that the photoperiodic control over *Kiss1* and *Rfrp* is indirect [see above, Ref. (50, 145, 161)]. In this context, a role for PT-derived TSH appeared plausible. In a landmark study, Klosen et al. (144) showed that intracerebroventricular delivery of TSH in Siberian and Syrian hamsters induces *Dio2* expression within ependymal cells, restores expression of *Kiss1* and *Rfrp* to their LP levels and, most importantly, triggers reactivation of the gonadal axis. Furthermore, Henson et al. (176) showed that T3 injections to SP-adapted Siberian hamsters reactivated the gonadotropic axis, thereby confirming prior data (see Section “An Overview” and Figure 1), but also led to LP-like levels of RF-amide peptides within the MBH.

Therefore, even though a theoretical possibility exists that another TSH-dependent – but T3-independent pathway – leads to seasonal changes of the reproductive axis, the most parsimonious model is one in which T3 action on RF-amide neurons link the photoperiodic production of TSH within the PT to the seasonal control of GnRH secretion.

## Conflict of Interest Statement

The authors declare that the research was conducted in the absence of any commercial or financial relationships that could be construed as a potential conflict of interest.
